# Knowledge, attitudes, and practices of gastric cancer patients toward nutritional therapy

**DOI:** 10.3389/fmed.2025.1433849

**Published:** 2025-03-10

**Authors:** Hui Yu, Ling Li, Jing Gu, Jing Wang, Hui Su, Hui Lu, Yuqing Zhou, Jingfang Xia, Yongping Xu, Danhua Liang, Yuling Yang, Ying Chen

**Affiliations:** ^1^Thoracic and Abdominal Radiotherapy Department, Affiliated Hospital of Jiangnan University, Wuxi, China; ^2^Obstetrics Department, Affiliated Hospital of Jiangnan University, Wuxi, China; ^3^Head and Neck Radiotherapy Department, Affiliated Hospital of Jiangnan University, Wuxi, China; ^4^Oncology Department 1, Affiliated Hospital of Jiangnan University, Wuxi, China; ^5^Oncology Department 2, Affiliated Hospital of Jiangnan University, Wuxi, China; ^6^Oncology Department 3, Affiliated Hospital of Jiangnan University, Wuxi, China; ^7^Comprehensive Radiotherapy Department, Affiliated Hospital of Jiangnan University, Wuxi, China; ^8^Chinese and Western Integrative Oncology Department, Affiliated Hospital of Jiangnan University, Wuxi, China; ^9^Oncology Department 4, Affiliated Hospital of Jiangnan University, Wuxi, China; ^10^Oncology Department, Affiliated Hospital of Jiangnan University, Wuxi, China

**Keywords:** knowledge, attitude, practice, gastric cancer, patients, nutritional support, cross-sectional study

## Abstract

**Background:**

To investigate the knowledge, attitude, and practice (KAP) of patients with gastric cancer (GC) toward nutritional therapy.

**Methods:**

This cross-sectional study was conducted from January to March 2024 at the Affiliated Hospital of Jiangnan University (Wuxi, China) and enrolled patients with GC. Questionnaires (Cronbach’s *α* = 0.923) were used to collect data on demographics and KAP dimensions. Scores >75% were considered good. Multivariable analyses were performed to examine the factors associated with KAP. A structural equation modeling (SEM) analysis was performed to examine the relationships among KAP dimensions.

**Results:**

The analysis included 486 valid questionnaires. The median knowledge, attitude, and practice scores were 6.0 (0–16; 37.5%), 26.0 (7–35; 74.3%), and 28.7 (8–40; 71.7%) indicating poor KAP. Only agricultural, forestry, animal husbandry, fishery, and water conservancy production personnel (OR = 0.09, 95%CI: 0.02–0.49, *p* = 0.006) were independently associated with knowledge. Knowledge (OR = 1.11, 95%CI: 1.05–1.18, *p* < 0.001) and a monthly income of 10,000–20,000 (OR = 3.85, 95%CI: 1.23–12.06, *p* = 0.021) were independently associated with attitude. Knowledge (OR = 1.22, 95%CI: 1.15–1.30, *p* < 0.001), attitude (OR = 1.21, 95%CI: 1.11–1.32, *p* < 0.001), personnel other than leading cadres of state organs and enterprises (all OR < 1 and all *p* < 0.05), and a monthly income of 10,000–20,000 yuan (OR = 3.02, 95%CI: 1.15–7.96, *p* = 0.025) were independently associated with practice. Knowledge had a direct positive influence on attitude (*β* = 0.350, *p* < 0.001) and practice (*β* = 0.460, *p* < 0.001) and an indirect positive influence on practice (*β* = 0.146, *p* < 0.001). Attitude had a direct positive influence on practice (*β* = 0.417, *p* < 0.001).

**Conclusion:**

Patients with GC in Wuxi partly had poor KAP toward nutritional support.

## Introduction

In recent years, the overall incidence of gastric cancer has been gradually declining worldwide, passing from the fifth most common cancer to the sixth position (1), but it remains a high-incidence cancer in China, with 43.9% of the global cases (2). Being a major player in the digestive system, removing the stomach (in part or totally) will compromise the digestive and nutritional functions of the patient (3, 4). Patients with advanced gastric cancer can also experience malnutrition preoperatively, compromising the perioperative period due to the decline in skeletal muscle mass and function, leading to increased rates of postoperative complications, nosocomial infections, mortality, prolonged hospitalization, decreased quality of life, and increased medical expenses (5, 6). In advanced stages, malnutrition in patients with gastric cancer can also lead to increased rates of adverse reactions during chemotherapy, decreased treatment tolerance, and reduced treatment completion rates, impacting treatment efficacy and quality of life and ultimately resulting in poorer survival (7). Factors such as inadequate nutrient intake, weight loss, and undergoing anti-tumor treatments (including surgery, radiotherapy, and systemic therapies) are considerations for selecting indications for nutritional intervention (7, 8). Currently, nutritional support for patients with gastric cancer aims primarily to preserve lean body tissue, reduce the occurrence of complications during the perioperative and peri-chemotherapy periods, ensure the safe completion of radical gastrectomy, ensure completion of adequate doses and courses of radiotherapy and chemotherapy, ultimately improving efficacy and prognosis, and realizing benefits from a health economics perspective (6, 9, 10).

In patients still able to feed by themselves, selecting healthy foods that will optimize energy and nutrient intake is paramount. For patients who will need nutritional support, having some knowledge about nutritional support and nutrition in the context of cancer could help the discussion with healthcare providers and translate into better choices and practice. Knowledge, attitude, and practice (KAP) surveys are research tools that provide quantitative and qualitative data about the gaps, misconceptions, and misunderstandings that can impair the optimal performance of a specific subject in a specific population (11, 12). KAP studies are particularly useful to design interventional and motivational interventions in a specific population. One study investigated the beliefs and experiences of patients with esophageal cancer toward nutritional support, but only in the peri-radiotherapy period (13). A recent study identified “caregiver self-efficacy and preparedness,” “caregiver needs are neglected,” and “nutrition as a source of conflict” as barriers to optimal nutrition in patients with gastric cancer (14). A systematic review highlighted that Chinese cancer patients have a poor KAP toward healthy eating (15). Nevertheless, the KAP of patients with gastric cancer regarding nutritional therapy remains unclear.

Hence, this study aimed to investigate the KAP of patients with gastric cancer toward nutritional therapy. The results could help design interventions to improve the KAP of patients toward nutritional status, which could translate into better patient outcomes.

## Methods

The study was reported according to the Checklist for Reporting Results of Internet E-Surveys (CHERRIES) (Supplementary material) (16).

### Study design and participants

This cross-sectional study was conducted from January to March 2024 at the Affiliated Hospital of Jiangnan University (Wuxi, China) and enrolled patients with gastric cancer through convenience sampling. This study was approved by the Medical Ethics Committee of the Affiliated Hospital of Jiangnan University (Ethic No. LS2023102). All participants provided written or online informed consent before completing the survey.

The inclusion criteria were (1) patients with gastric cancer who have undergone surgery and (2) agreed to participate in the study. The exclusion criteria were (1) severe cognitive impairment, (2) mental abnormalities, or (3) any conditions that prevent normal communication or ability to complete a questionnaire.

### Questionnaire

The design of the questionnaire was based on guidelines on nutritional support in cancer patients (17–20) and relevant literature (21). After the initial design, feedback was sought from five senior oncology nursing experts, six experts in oncology nursing and nursing management, and three associate chief oncologists. The questionnaire was subsequently revised based on their suggestions and underwent a pilot study with a small sample size (23 respondents), resulting in a reliability coefficient (Cronbach’s *α*) of 0.923.

The final questionnaire was in Chinese and consisted of four sections: demographic information (age, gender, residence, education level, occupation type, average monthly household income, marital status, medical insurance type, cancer classification, etc.), knowledge dimension, attitude dimension, and practice dimension. The knowledge dimension comprised two aspects with a total of eight questions, with 2 points for “very knowledgeable,” 1 point for “heard about it,” and 0 points for “not sure,” with a score range of 0–16 points. The attitude dimension consisted of seven questions scored using a 5-point Likert scale ranging from “strongly agree” (5 points) to “strongly disagree” (1 point), with a score range of 7–35 points. The practice dimension includes eight questions, which are also scored using a 5-point Likert scale ranging from “always” (5 points) to “never” (1 point), with a score range of 8–40 points. Adequate knowledge, positive attitude, and proactive practice were defined as a total score of each dimension >75% (22).

### Questionnaire distribution and quality control

This study combined online questionnaire filling and paper questionnaires. Participation in the study was voluntary, and no incentives were offered. Except for the modes of administration and responding, the two questionnaires were exactly the same, differing only in the mode of administration and response collection. All items were listed one after the other online and on the paper-based questionnaire. Paper questionnaires were distributed to the participants during inpatient and outpatient visits. The online questionnaire was distributed via Questionnaire Star^1^ to the participants. The participants could scan the QR code using WeChat or follow the provided link to access and complete the questionnaire. The survey was closed and limited to those receiving the QR code or obtaining a paper copy. The participants were assured of anonymity during the survey process. All data are stored on the secure servers at the corresponding author’s center. The research team was comprised of three nurses trained as research assistants. They were responsible for questionnaire promotion and distribution and the meticulous review of all submissions for completeness, internal consistency, and logical coherence. The investigators were trained to grasp the problem’s meaning and the investigation process, enhancing data accuracy and consistency. Responses to all items were mandatory for submission of the online questionnaire. The questionnaires containing incomplete answers (paper questionnaires missing one or more responses), questionnaires with uniform responses across all items, and questionnaires with all the knowledge items responded with “not sure” (which could raise doubt the questionnaire was read or answered carefully) were considered invalid.

### Sample size calculation

The sample size should ideally be at least 10 times the number of predictors (23). With 23 independent variables in this questionnaire, the minimum sample size required would be 230. Accounting for a 20% non-response rate, the final necessary sample size would be 288.

### Statistical analysis

The variables were tested for normal distribution using the Kolmogorov–Smirnov test. Normally distributed continuous data were presented as means ± standard deviations and analyzed using Student’s t-test (two-level comparisons) or ANOVA (comparison of more than two levels). Continuous data with a skewed distribution were presented as medians (interquartile ranges) and analyzed using the Mann-Whiney U-test (two-level comparisons) of the Kruskall-Wallis H-test (comparisons of more than two levels). Categorical data were presented as n (%). The correlations between KAP dimension scores were assessed using the Pearson correlation coefficient for data that meet the assumptions of normal distribution and the Spearman correlation coefficient for data that do not. Variables with a univariable *p*-value <0.1 were included in the multivariable analyses. Incorporating the KAP theoretical framework, a structural equation model (SEM) was used to verify whether attitudes mediate the relationship between knowledge and behavioral practices. The indirect and direct effects were calculated and compared. The threshold criteria for goodness-of-fit indices of the SEM model were RMSEA < 0.08, SRMR < 0.08, TLI > 0.8, and CFI > 0.8. If the goodness-of-fit thresholds cannot be met, path analysis was conducted to test the mediation effects. Statistical analysis was performed using Stata 18.0 (StataCorp LLC, College Station, TX, USA). *p*-values were reported to three decimals, and two-sided *p*-values <0.05 were considered statistically significant.

## Results

### Characteristics of the participants

In this study, 503 questionnaires were distributed (301 online and 202 paper), but 17 were considered invalid (all knowledge items answered with “not sure”). Hence, this analysis included 486 valid questionnaires. There were 291 (59.9%) males; 23.5, 31.9, and 29.4% of the participants were 50–59, 60–69, and ≥70 years old, respectively. The highest frequencies were observed for urban residents (59.5%), junior high school and below education (64.2%), ordinary staff and related personnel (33.3%), family monthly income of 5,000–10,000 CNY (37.4%), married (92.0%), and with medical insurance (98.8%) (Table 1).

**Table 1:** Characteristics of the participants.

*N* = 486	*n* (%)	Knowledge	*p*	Attitude	*p*	Practice	*p*
		Median		Median		Median	
**Total score**	486 (100.0)	6.00 [4.00, 9.75]	**<0.001**	26.00 [24.00, 28.00]	**<0.001**	28.66 (6.21)	0.105
**Gender**			0.136		0.432		0.647
Male	291 (59.9)	6.00 [4.00, 9.00]		26.00 [24.00, 27.00]		29.00 [24.00, 33.00]	
Female	195 (40.1)	7.00 [4.00, 10.00]		26.00 [24.00, 28.00]		29.00 [24.00, 33.00]	
**Age**			**0.006**		0.292		0.552
18–39	38 (7.8)	8.50 [7.00, 11.75]		27.00 [25.00, 29.00]		29.00 [24.00, 32.00]	
40–49	36 (7.4)	6.00 [5.00, 8.00]		26.00 [24.00, 29.00]		29.00 [25.00, 35.00]	
50–59	114 (23.5)	6.00 [4.00, 9.00]		27.00 [24.00, 27.00]		29.50 [24.25, 34.00]	
60–69	155 (31.9)	6.00 [4.00, 9.50]		26.00 [24.00, 27.00]		29.00 [24.00, 32.00]	
≥70	143 (29.4)	6.00 [3.50, 8.00]		26.00 [24.00, 28.00]		29.00 [24.00, 33.00]	
**Residence**			**0.038**		0.099		**0.002**
Rural	144 (29.6)	6.00 [4.00, 8.00]		26.00 [24.00, 27.00]		27.00 [23.00, 32.00]	
Urban	289 (59.5)	6.00 [4.00, 10.00]		26.00 [24.00, 28.00]		30.00 [25.00, 34.00]	
Suburban	53 (10.9)	6.00 [3.00, 10.00]		25.00 [23.00, 27.00]		26.00 [21.00, 32.00]	
**Education**			**<0.001**		0.076		0.617
Junior high school and below	312 (64.2)	6.00 [4.00, 8.00]		26.00 [24.00, 27.00]		29.00 [24.00, 33.00]	
High school/technical school	93 (19.1)	6.00 [4.00, 10.00]		27.00 [24.00, 28.00]		29.00 [25.00, 33.00]	
College	39 (8.0)	8.00 [4.50, 12.00]		27.00 [25.00, 28.00]		29.00 [25.00, 32.00]	
Bachelor’s degree	38 (7.8)	8.00 [6.00, 10.75]		27.00 [25.00, 28.00]		29.00 [25.00, 36.25]	
Master’s degree and above	4 (0.8)	12.00 [9.75, 14.50]		29.00 [26.75, 31.00]		28.50 [26.00, 31.75]	
**Occupation type**			**0.001**		**0.005**		**<0.001**
Leading cadres of state organs and enterprises	39 (8.0)	6.00 [5.00, 11.50]		27.00 [26.00, 28.50]		34.00 [30.50, 36.00]	
Professional and technical personnel	43 (8.8)	9.00 [6.00, 12.00]		26.00 [24.50, 28.50]		29.00 [24.50, 33.00]	
Ordinary staff and related personnel	162 (33.3)	6.50 [4.25, 10.00]		26.00 [24.00, 28.00]		28.00 [24.00, 32.00]	
Business and service personnel	15 (3.1)	6.00 [4.50, 10.00]		27.00 [26.00, 28.00]		34.00 [29.00, 36.50]	
Agricultural, forestry, animal husbandry, fishery, and water conservancy production personnel	57 (11.7)	5.00 [4.00, 7.00]		25.00 [24.00, 28.00]		27.00 [22.00, 32.00]	
Production and transportation equipment operators	12 (2.5)	6.00 [3.00, 8.25]		26.50 [23.50, 27.25]		32.00 [21.75, 33.25]	
Military personnel	23 (4.7)	7.00 [4.00, 9.00]		27.00 [24.00, 28.00]		27.00 [23.50, 31.50]	
Other	135 (27.8)	6.00 [4.00, 8.50]		25.00 [24.00, 27.00]		28.00 [24.00, 32.00]	
**Family monthly income**			0.711		**0.005**		**<0.001**
<2,000	62 (12.8)	7.50 [4.00, 11.00]		26.00 [24.00, 27.00]		26.50 [24.00, 32.00]	
2,000–5,000	171 (35.2)	6.00 [4.00, 9.00]		26.00 [24.00, 27.00]		27.00 [23.00, 32.00]	
5,000–10,000	182 (37.4)	6.00 [4.00, 8.75]		26.00 [24.00, 27.75]		29.00 [25.00, 33.00]	
10,000–20,000	57 (11.7)	6.00 [5.00, 10.00]		27.00 [26.00, 29.00]		32.00 [28.00, 35.00]	
>20,000	14 (2.9)	7.00 [4.25, 9.00]		25.00 [23.25, 30.25]		26.50 [24.00, 31.25]	
**Marital status**			0.904		0.285		0.117
Single	14 (2.9)	6.50 [4.25, 12.25]		25.50 [24.00, 28.50]		24.50 [23.25, 37.00]	
Married	447 (92.0)	6.00 [4.00, 9.00]		26.00 [24.00, 28.00]		29.00 [24.00, 33.00]	
Divorced	9 (1.9)	7.00 [2.00, 14.00]		26.00 [25.00, 29.00]		31.00 [26.00, 32.00]	
Widowed	16 (3.3)	6.00 [2.75, 9.25]		25.00 [23.75, 26.25]		24.50 [20.75, 30.00]	
**Medical insurance**			0.166		**0.031**		**0.031**
Only social medical insurance	440 (90.5)	6.00 [4.00, 9.00]		26.00 [24.00, 28.00]		29.00 [24.00, 33.00]	
Only commercial medical insurance							
Both social insurance and commercial insurance	40 (8.2)	8.00 [5.00, 12.25]		27.00 [25.00, 28.00]		30.00 [24.75, 38.25]	
No insurance	6 (1.2)	4.50 [2.50, 10.25]		23.00 [22.00, 26.25]		24.50 [21.75, 25.75]	

Bold indicates statistically significant differences.

### Knowledge

The median knowledge score was 6.0 (0–16; 37.5%), indicating poor knowledge. Differences in knowledge were observed according to age (*p* = 0.006), residence (*p* = 0.038), education (*p* < 0.001), and occupation (*p* = 0.001) (Table 1). The knowledge item with the highest score was K1 (25.9% very familiar; 61.5% heard of; “Malnutrition is common among gastric cancer patients, and compared to other tumors, gastric cancer is more prone to causing severe and prolonged malnutrition.”). The item with the lowest score was K4 (11.9% very familiar; 33.3% heard of; “During surgery, the energy intake goal for patients is measured based on actual measurements or calculated at 104.65 to 125.58 kilojoules per kilogram of body weight, and the protein intake goal is 1.2 to 1.5 grams per kilogram of body weight.”) (Table 2).

**Table 2:** Knowledge.

Knowledge	*n* (%)		
	Very familiar	Heard of	Not sure
K1. Malnutrition is common among gastric cancer patients, and compared to other tumors, gastric cancer is more prone to causing severe and prolonged malnutrition.	126 (25.9)	299 (61.5)	61 (12.6)
K2. Malnutrition in gastric cancer patients can lead to increased postoperative complications, higher risks of infection and complications, reduced quality of life, and increased medical expenses.	116 (23.9)	275 (56.6)	95 (19.5)
K3. The goal of nutritional support is to maintain weight, reduce complications, and ensure the effectiveness of surgery and treatment.	122 (25.1)	268 (55.1)	96 (19.8)
K4. During surgery, the energy intake goal for patients is measured based on actual measurements or calculated at 104.65 to 125.58 kilojoules per kilogram of body weight, and the protein intake goal is 1.2 to 1.5 grams per kilogram of body weight.	58 (11.9)	162 (33.3)	266 (54.7)
K5. It is recommended to use immunonutrition during the perioperative period, including arginine, glutamine, omega-3 fatty acids, and nucleotides, for a duration of 5 to 7 days.	65 (13.4)	177 (36.4)	244 (50.2)
K6. The sequence of nutritional therapy includes nutritional counseling, oral nutritional supplements, enteral nutrition, and parenteral nutrition, with each level initiated as needed.	93 (19.1)	220 (45.3)	173 (35.6)
K7. Patients who undergo subtotal gastrectomy should regularly monitor levels of vitamin B12, folate, iron, calcium, and vitamin D, and supplement deficiencies accordingly.	75 (15.4)	244 (50.2)	167 (34.4)
K8. Patients after gastric cancer surgery should consider prophylactic supplementation of calcium and vitamin D, and increase intake of calcium-rich foods such as milk, cheese, sardines, and dark green vegetables.	113 (23.3)	287 (59.1)	86 (17.7)

### Attitude

The median attitude score was 26.0 (7–35; 74.3%), indicating a negative attitude. Differences in attitude were observed according to occupation (*p* = 0.005), income (*p* = 0.005), and insurance (*p* = 0.031) (Table 1). The attitude item with the highest score was A2 (93.9% agree; “During the perioperative and peri-chemotherapy periods, I need to pay special attention to my nutritional intake to reduce complications and improve treatment outcomes.”). The item with the lowest score was A7 (60.5% agree; “I am worried that nutritional therapy will increase my financial burden, and I am unsure if I can afford the associated costs.”) (Table 3).

**Table 3:** Attitude.

Attitude	Strongly agree	Agree	Neutral	Disagree	Strongly disagree
A1. Malnutrition will have negative effects on my health, so I need to pay attention to and improve my nutritional status.	200 (41.2)	247 (50.8)	37 (7.6)	1 (0.2)	1 (0.2)
A2. During the perioperative and peri-chemotherapy periods, I need to pay special attention to my nutritional intake to reduce complications and improve treatment outcomes.	221 (45.5)	235 (48.4)	29 (6)	1 (0.2)	0 (0)
A3. I need to determine my intake goals reasonably based on my weight and actual situation.	202 (41.6)	224 (46.1)	55 (11.3)	5 (1)	0 (0)
A4. I am concerned about nutritional therapy and worry that it will restrict my freedom to enjoy food.	127 (26.1)	178 (36.6)	123 (25.3)	50 (10.3)	8 (1.6)
A5. I hope healthcare professionals can provide me with more education and opportunities for learning and consultation to improve my nutritional knowledge.	226 (46.5)	208 (42.8)	50 (10.3)	1 (0.2)	1 (0.2)
A6. I am confident that I can follow the advice of doctors and nutritionists, make appropriate dietary choices for myself, and develop good nutritional habits.	211 (43.4)	215 (44.2)	58 (11.9)	1 (0.2)	1 (0.2)
A7. I am worried that nutritional therapy will increase my financial burden, and I am unsure if I can afford the associated costs.	105 (21.6)	189 (38.9)	130 (26.7)	51 (10.5)	11 (2.3)

### Practice

The median practice score was 28.7 (8–40; 71.7%), indicating poor practice. Differences in practice were observed according to residence (*p* = 0.002), occupation (*p* < 0.001), income (*p* < 0.001), and insurance (*p* = 0.031) (Table 1). The practice item with the highest score was P3 (74.3% consistent; “I will choose foods rich in vitamins and minerals, such as fresh fruits, vegetables, whole grains, and low-fat dairy products, to support immune system function and overall health.”). The item with the lowest score was P7 (39.9% consistent; “I will actively participate in nutritional education activities to enhance my knowledge of nutritional therapy.”) (Table 4).

**Table 4:** Practice.

Practice	Very consistent	Somewhat consistent	Neutral	Somewhat inconsistent	Very inconsistent
P1. I will regularly keep track of my diet to understand my nutritional intake.	72 (14.8)	138 (28.4)	128 (26.3)	82 (16.9)	66 (13.6)
P2. I will increase my consumption of high-protein foods such as fish, poultry, legumes, and nuts to help maintain muscle mass and support recovery.	158 (32.5)	200 (41.2)	107 (22)	16 (3.3)	5 (1)
P3. I will choose foods rich in vitamins and minerals, such as fresh fruits, vegetables, whole grains, and low-fat dairy products, to support immune system function and overall health.	169 (34.8)	192 (39.5)	107 (22)	12 (2.5)	6 (1.2)
P4. I will regularly monitor my nutritional status and supplement as needed with vitamins B12, folate, iron, calcium, and others.	77 (15.8)	119 (24.5)	136 (28)	106 (21.8)	48 (9.9)
P5. Before and after surgery, I will follow the advice of doctors and nutritionists to prepare for and recover from the necessary nutritional support to improve the success rate of the surgery and expedite the recovery process.	139 (28.6)	204 (42)	111 (22.8)	25 (5.1)	7 (1.4)
P6. I will maintain adequate fluid intake to prevent dehydration and aid in digestion and waste elimination.	162 (33.3)	190 (39.1)	110 (22.6)	22 (4.5)	2 (0.4)
P7. I will actively participate in nutritional education activities to enhance my knowledge of nutritional therapy.	69 (14.2)	125 (25.7)	145 (29.8)	111 (22.8)	36 (7.4)
P8. I will supplement with vitamin tablets to meet my body’s vitamin requirements, especially when sufficient amounts cannot be obtained through diet alone.	80 (16.5)	149 (30.7)	122 (25.1)	79 (16.3)	56 (11.5)

### Correlations

As shown in Table 5, the knowledge scores correlated to the attitude (*r* = 0.174, *p* < 0.001) and practice (*r* = 0.387, *p* < 0.001) scores, while the attitude scores correlated to the practice scores (*r* = 0.374, *p* < 0.001).

**Table 5:** Correlation analysis.

	Knowledge	Attitude	Practice
Knowledge	1.000		
Attitude	0.174 (*p* < 0.001)	1.000	
Practice	0.387 (*p* < 0.001)	0.374 (*p* < 0.001)	1.000

### Multivariable analyses

Only agricultural, forestry, animal husbandry, fishery, and water conservancy production personnel (OR = 0.09, 95%CI: 0.02–0.49, *p* = 0.006) were independently associated with knowledge (Table 6). The knowledge scores (OR = 1.11, 95%CI: 1.05–1.18, *p* < 0.001) and a monthly income of 10,000–20,000 yuan (OR = 3.85, 95%CI: 1.23–12.06, *p* = 0.021) were independently associated with the attitude scores (Table 7). The knowledge scores (OR = 1.22, 95%CI: 1.15–1.30, *p* < 0.001), the attitude scores (OR = 1.21, 95%CI: 1.11–1.32, *p* < 0.001), professional and technical personnel (OR = 0.21, 95%CI: 0.06–0.73, *p* = 0.014), ordinary staff and related personnel (OR = 0.25, 95%CI: 0.08–0.75, *p* = 0.013), military personnel (OR = 0.22, 95%CI: 0.06–0.88, *p* = 0.032), other personnel (OR = 0.31, 95%CI: 0.10–0.95, *p* = 0.040), and a monthly income of 10,000–20,000 (OR = 3.02, 95%CI: 1.15–7.96, *p* = 0.025) were independently associated with the practice scores (Table 8).

**Table 6:** Univariable and multivariable analyses of knowledge.

Knowledge (≥12)	Univariable analysis	*p*	Multivariable analysis	*p*
	OR (95%CI)		OR (95%CI)	
**Gender**				
Male				
Female	1.21 (0.74, 1.94)	0.444		
**Age**				
18–39				
40–49	0.35 (0.09, 1.17)	0.104	0.59 (0.14, 2.45)	0.466
50–59	0.46 (0.19, 1.15)	0.086	0.77 (0.24, 2.50)	0.661
60–69	0.62 (0.27, 1.47)	0.255	1.02 (0.33, 3.13)	0.974
≥70	0.56 (0.25, 1.36)	0.185	1.04 (0.34, 3.25)	0.942
**Residence**				
Rural				
Urban	1.65 (0.94, 2.99)	0.089	1.41 (0.72, 2.79)	0.319
Suburban	1.43 (0.58, 3.35)	0.419	1.26 (0.50, 3.20)	0.622
**Education**				
Junior high school and below				
High school/technical school	1.27 (0.66, 2.33)	0.461	0.97 (0.49, 1.93)	0.932
College	2.39 (1.07, 5.04)	**0.026**	1.52 (0.60, 3.82)	0.376
Bachelor’s degree	1.89 (0.8, 4.13)	0.125	1.33 (0.42, 4.21)	0.631
Master’s degree and above	6.09 (0.72, 51.84)	0.075	2.59 (0.23, 29.15)	0.442
**Occupation type**				
Leading cadres of state organs and enterprises				
Professional and technical personnel	1.26 (0.48, 3.38)	0.644	1.06 (0.37, 3.05)	0.914
Ordinary staff and related personnel	0.58 (0.26, 1.38)	0.198	0.58 (0.23, 1.47)	0.252
Business and service personnel	1.05 (0.25, 3.93)	0.939	0.94 (0.23, 3.88)	0.927
Agricultural, forestry, animal husbandry, fishery, and water conservancy production personnel	0.11 (0.02, 0.43)	**0.005**	0.09 (0.02, 0.49)	**0.006**
Production and transportation equipment operators	0.26 (0.01, 1.63)	0.228	0.27 (0.03, 2.57)	0.254
Military personnel	0.81 (0.22, 2.66)	0.729	0.77 (0.20, 2.99)	0.705
Other	0.5 (0.22, 1.23)	0.119	0.45 (0.17, 1.24)	0.124
**Family monthly income**				
<2,000				
2,000–5,000	0.51 (0.25, 1.07)	0.070	0.37 (0.16, 0.82)	
5,000–10,000	0.64 (0.32, 1.32)	0.215	0.37 (0.16, 0.83)	
10,000–20,000	0.67 (0.27, 1.62)	0.375	0.31 (0.11, 0.91)	
>20,000	0.52 (0.08, 2.21)	0.428	0.17 (0.03, 1.14)	
**Marital status**				
Single				
Married	0.49 (0.16, 1.82)	0.236		
Divorced	1.25 (0.19, 7.77)	0.809		
Widowed	0.36 (0.04, 2.2)	0.283		
**Type of medical insurance**				
Only social medical insurance				
Only commercial medical insurance				
Both social insurance and commercial insurance	2.04 (0.94, 4.17)	0.059	1.76 (0.77, 4.00)	0.177
No insurance	2.69 (0.37, 14.05)	0.259	3.50 (0.47, 26.11)	0.222

Bold indicates statistically significant differences.

**Table 7:** Univariable and multivariable analyses of attitude.

Attitude (≥25)	Univariable analysis	*p*	Multivariable analysis	*p*
	OR (95%CI)		OR (95%CI)	
**Knowledge score**	1.12 (1.06, 1.19)	**<0.001**	1.11 (1.05, 1.18)	**<0.001**
**Gender**				
Male				
Female	1.03 (0.69, 1.55)	0.874		
**Age**				
18–39				
40–49	0.59 (0.19, 1.74)	0.342	0.63 (0.19, 2.12)	0.460
50–59	0.53 (0.20, 1.27)	0.175	0.57 (0.21, 1.56)	0.271
60–69	0.63 (0.24, 1.46)	0.308	0.80 (0.30, 2.17)	0.664
≥70	0.46 (0.18, 1.07)	0.089	0.59 (0.22, 1.61)	0.305
**Residence**				
Rural				
Urban	1.06 (0.67, 1.64)	0.803		
Suburban	0.71 (0.37, 1.4)	0.320		
**Education**				
Junior high school and below				
High school/technical school	1.25 (0.75, 2.12)	0.405		
College	1.77 (0.82, 4.27)	0.167		
Bachelor’s degree	1.72 (0.79, 4.14)	0.194		
Master’s degree and above	2,636,755.42 (0, NA)	0.984		
**Occupation type**				
Leading cadres of state organs and enterprises				
Professional and technical personnel	0.43 (0.12, 1.31)	0.152	0.41 (0.12, 1.44)	0.162
Ordinary staff and related personnel	0.42 (0.14, 1.06)	0.090	0.54 (0.18, 1.59)	0.261
Business and service personnel	2.06 (0.3, 41.34)	0.527	2.76 (0.28, 27.13)	0.385
Agricultural, forestry, animal husbandry, fishery, and water conservancy production personnel	0.23 (0.07, 0.65)	**0.008**	0.41 (0.13, 1.34)	0.142
Production and transportation equipment operators	0.29 (0.06, 1.41)	0.115	0.58 (0.11, 2.92)	0.504
Military personnel	0.34 (0.09, 1.21)	0.098	0.51 (0.13, 2.02)	0.337
Other	0.28 (0.09, 0.69)	**0.012**	0.38 (0.13, 1.14)	0.084
**Family monthly income**				
<2,000				
2,000–5,000	1.04 (0.54, 1.94)	0.904	1.05 (0.53, 2.08)	0.892
5,000–10,000	0.97 (0.51, 1.79)	0.921	0.88 (0.44, 1.75)	0.721
10,000–20,000	4.6 (1.69, 14.78)	**0.005**	3.85 (1.23, 12.06)	**0.021**
>20,000	0.44 (0.13, 1.46)	0.174	0.33 (0.09, 1.22)	0.098
**Marital status**				
Single				
Married	1.42 (0.43, 4.18)	0.541		
Divorced	1.94 (0.31, 16.56)	0.496		
Widowed	0.93 (0.20, 4.15)	0.919		
**Type of medical insurance**				
Only social medical insurance				
Only commercial medical insurance				
Both social insurance and commercial insurance	1.62 (0.76, 3.87)	0.236	1.57 (0.65, 3.78)	0.313
No insurance	0.20 (0.03, 1.05)	0.067	0.19 (0.03, 1.19)	0.076

Bold indicates statistically significant differences.

**Table 8:** Univariable and multivariable analyses of practice.

Practice (≥28)	Univariable analysis	*p*	Multivariable analysis	*p*
	OR (95%CI)		OR (95%CI)	
**Knowledge**	1.19 (1.12, 1.27)	**<0.001**	1.22 (1.15, 1.30)	**<0.001**
**Attitude**	1.24 (1.14, 1.35)	**<0.001**	1.21 (1.11, 1.32)	**<0.001**
**Gender**				
Male				
Female	1.16 (0.80, 1.68)	0.426		
**Age**				
18–39				
40–49	1.3 (0.51, 3.42)	0.584		
50–59	0.83 (0.39, 1.75)	0.636		
60–69	0.86 (0.41, 1.75)	0.675		
≥70	0.76 (0.36, 1.57)	0.462		
**Residence**				
Rural				
Urban	1.9 (1.27, 2.86)	**0.002**	1.58 (0.95, 2.62)	0.078
Suburban	0.83 (0.44, 1.57)	0.573	0.77 (0.36, 1.65)	0.507
**Education**				
Junior high school and below				
High school/technical school	1.28 (0.8, 2.06)	0.303		
College	1.22 (0.62, 2.43)	0.570		
Bachelor’s degree	1.63 (0.82, 3.39)	0.177		
Master’s degree and above	2.54 (0.32, 51.62)	0.422		
**Occupation type**				
Leading cadres of state organs and enterprises				
Professional and technical personnel	0.22 (0.07, 0.65)	**0.009**	0.21 (0.06, 0.73)	**0.014**
Ordinary staff and related personnel	0.18 (0.06, 0.44)	**0.001**	0.25 (0.08, 0.75)	**0.013**
Business and service personnel	0.96 (0.18, 7.25)	0.960	1.63 (0.25, 10.76)	0.610
Agricultural, forestry, animal husbandry, fishery, and water conservancy production personnel	0.14 (0.04, 0.39)	**<0.001**	0.45 (0.13, 1.53)	0.201
Production and transportation equipment operators	0.21 (0.04, 0.91)	0.037	0.58 (0.10, 3.27)	0.540
Military personnel	0.13 (0.04, 0.45)	**0.002**	0.22 (0.06, 0.88)	**0.032**
Other	0.15 (0.05, 0.37)	**<0.001**	0.31 (0.10, 0.95)	**0.040**
**Family monthly income**				
<2,000				
2,000–5,000	1.1 (0.61, 1.97)	0.751	1.22 (0.62, 2.42)	0.567
5,000–10,000	1.78 (1, 3.2)	0.052	1.99 (0.98, 4.02)	0.055
10,000–20,000	4.27 (1.94, 9.87)	**<0.001**	3.02 (1.15, 7.96)	**0.025**
>20,000	1.14 (0.35, 3.7)	0.827	1.26 (0.30, 5.22)	0.750
**Marital status**				
Single				
Married	1.82 (0.62, 5.61)	0.275		
Divorced	2.67 (0.49, 17.18)	0.270		
Widowed	0.8 (0.18, 3.5)	0.765		
**Type of medical insurance**				
Only social medical insurance				
Only commercial medical insurance				
Both social insurance and commercial insurance	1.13 (0.59, 2.22)	0.718	0.83 (0.38, 1.82)	0.647
No insurance	0.15 (0.01, 0.94)	0.085	0.19 (0.02, 2.02)	0.168

Bold indicates statistically significant differences.

### Structural equation modeling

**Figure 1:**
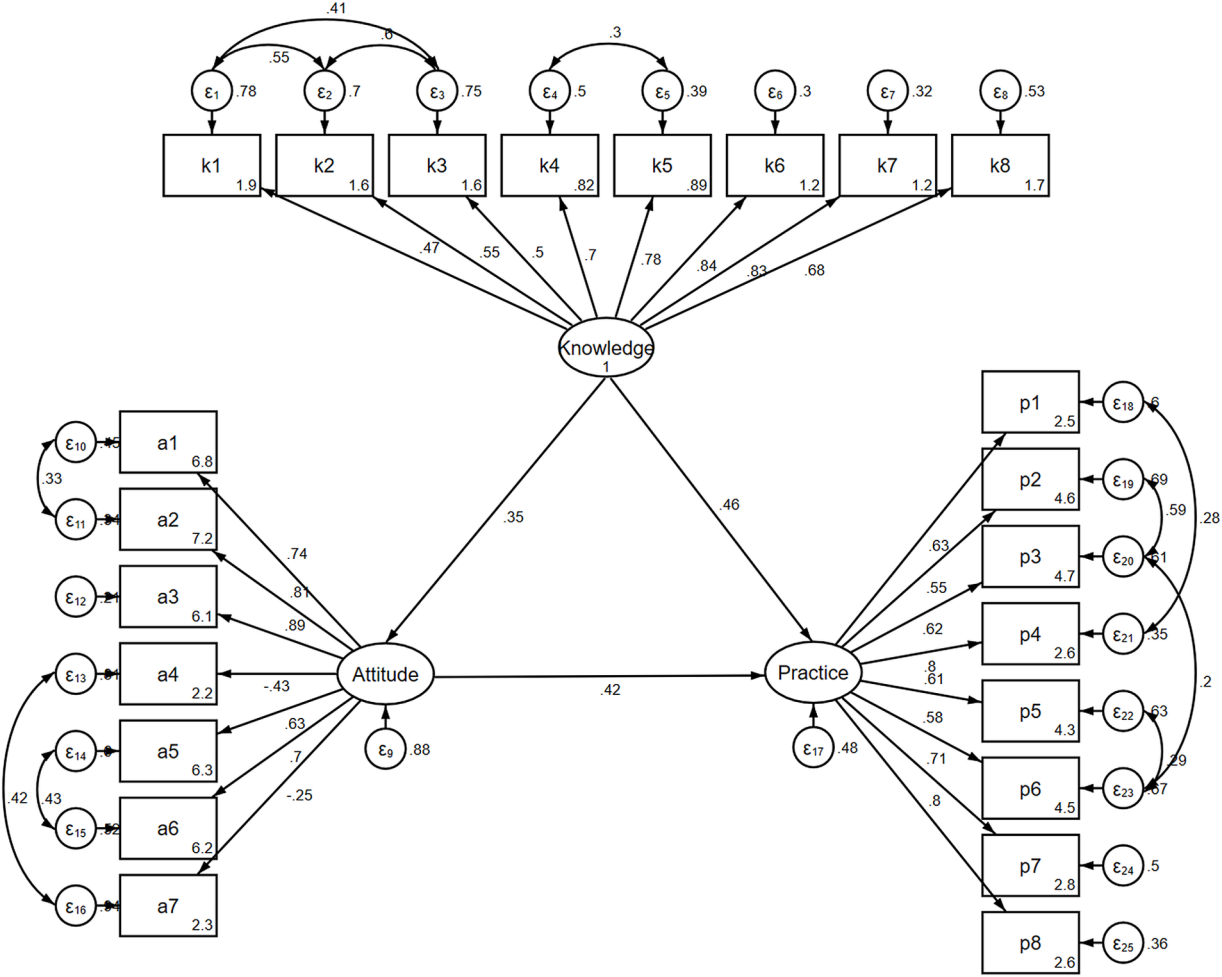
Structural equation modeling.

The SEM is depicted in Figure 1. All four SEM fit indices showed good fit (Table 9). Knowledge had a direct positive influence on attitude (*β* = 0.350, *p* < 0.001) and practice (*β* = 0.460, *p* < 0.001) and an indirect positive influence on practice (*β* = 0.146, *p* < 0.001). Attitude had a direct positive influence on practice (*β* = 0.417, *p* < 0.001) (Table 10).

**Table 9:** SEM fit indicators.

Indicators	Reference	Results
RMSEA	<0.08 is good	0.073
SRMR	<0.08 is good	0.078
TLI	>0.8 is good	0.896
CFI	>0.8 is good	0.911

**Table 10:** SEM analysis.

Model paths		Total effects		Direct effect		Indirect effect	
		*β* (95%CI)	*p*	*β* (95%CI)	*p*	*β* (95%CI)	*p*
Asum							
	Ksum	0.350 (0.260, 0.440)	<0.001	0.350 (0.260, 0.440)	<0.001		
Psum							
	Asum	0.417 (0.333, 0.501)	<0.001	0.417 (0.333, 0.501)	<0.001		
	Ksum	0.606 (0.535, 0.677)	<0.001	0.460 (0.379, 0.541)	<0.001	0.146 (0.100, 0.192)	<0.001

## Discussion

Proper nutritional support is crucial in cancer patients due to the physiological stresses of the cancer, surgery, radiotherapy, and chemotherapy (19). Nutritional support in patients with GC is particularly important due to the impact of the cancer and then gastrectomy on digestion and nutrition (3, 4). A proper nutritional status is conducive to a better prognosis (6, 9, 10). Still, maintaining a proper nutritional status requires knowledge and attitude to make adequate lifestyle choices and be able to make informed choices about medical nutritional support. The present study of patients with gastric cancer in Wuxi indicates that the KAP toward nutritional support is poor. No previous studies examined the question in patients with GC, but a previous study of patients with esophageal cancer in the peri-radiotherapy period showed that the lack of nutrition-related knowledge, a lack of motivation, and factors related to nutrition were the main barriers to maintaining a proper nutritional status, supporting the present study. Tang et al. (15) also reported that Chinese cancer patients had a poor KAP toward healthy eating. Although nutritional support might be perceived as secondary to cancer therapies by the patients, healthcare providers should provide adequate information about nutrition to the patients. Still, a study showed that digestive surgeons had poor KAP toward nutritional support to cancer patients (24). Although the KAP of physicians was not assessed in the present study, it should be investigated in the future to design continuing education activities to improve the KAP of physicians. Indeed, healthcare providers are widely regarded as crucial sources of reliable health-related knowledge by patients (25, 26). Poor knowledge in the physicians could lead to inaccurate information being transferred to the patients.

The correlation, multivariable, and SEM analyses showed that knowledge influenced attitude and practice, and attitude influenced practice. Hence, improving knowledge through education activities should translate into better practice, as supported by the KAP theory (11, 12, 27). The KAP conceptual framework considers knowledge to be the basis for practice and that attitudes are the force driving practice (11, 12, 27). Hence, improving knowledge should lead to more positive attitudes and more proactive practices.

The multivariable analyses revealed a positive association between superior job positions and higher income levels with better KAP. It is widely acknowledged that individuals with higher socioeconomic status tend to possess greater healthcare literacy (28). Therefore, it is imperative to meticulously screen patients and implement tailored educational interventions to enhance healthcare outcomes. The present study suggests that patients with GC with a lower income and lower job position could be the ones most in need of proper education about nutrition in GC.

Translating KAP findings into actionable recommendations is an important step in maximizing the impact of such studies. The KAP conceptual framework assumes that improving knowledge can lead to more positive attitudes and proactive practices (11, 12, 27). Based on its findings, the present study identified specific knowledge gaps and demographic groups that could benefit from targeted interventions. All eight knowledge items showed poor scores, highlighting the need for education of patients with GC regarding nutritional support, including the risk of malnutrition in GC, the goal of nutritional support, the content and administration of nutritional support in patients with GC, and the need for the supplementation of specific micronutrients and vitamins. In addition, knowledge scores were lower in individuals not having desk occupations, which are often associated with a lower socioeconomic status. The results could inform the design of a nutritional information and education campaign tailored to the specific needs of the population in Wuxi City, focusing on agricultural, forestry, animal husbandry, fishery, and water conservancy production personnel and individuals with a lower income to highlight knowledge about the risk of malnutrition in GC, the health hazards associated with malnutrition in the oncological context, the goal of malnutrition, the proper energy intake, proper nutrients after surgery, the types of nutrition after surgery, and the prophylactic use of specific vitamins and micronutrients. On a policy level, guidelines and consensuses for nutritional support in patients with gastric cancer are available (6, 9, 10, 29), and policymakers and stakeholders should be aware of such guidelines and provide support and resources to the physicians and patients to help them implement optimal nutritional support after gastric cancer. Teaching patients what and how to eat after gastric cancer is important, but some patients could require more support to achieve optimal outcomes. Various support programs are available in different provinces and countries. Nutritional support programs for patients with gastric cancer can help with eating difficulties, weight loss, and side effects. Programs may include nutrition counseling, adapted recipes, and cooking demonstrations (6, 9, 10, 30, 31).

This study had limitations. It was a single-center study that enrolled participants from a single geographical area, limiting generalizability. The study was cross-sectional, preventing the analysis of causality. The participants were selected through convenience sampling, which could introduce bias, and future studies should consider probability sampling. A SEM analysis was performed to examine causality, but causality is statistically inferred instead of being observed, and the results must be taken cautiously (32–34). In addition, the data represent a single point in time, but they could serve as a historical control to examine the effects of future interventions on KAP. All KAP studies are at risk of social desirability bias, according to which the participants can be tempted to answer what they know they should think and do instead of what they actually do (35, 36). Considering that all KAP scores were poor, that bias is less likely. All data were self-reported. Since the accuracy of self-reported clinical data relies directly on the degree of understanding of the patient regarding his/her condition, precise clinical data were not collected from the patients because of the high risks of various biases. In addition, linking the questionnaire to the patient’s chart was not possible because the questionnaires were completed anonymously. Therefore, including the exact condition of the patients in the analysis was not possible. In order to accommodate as many participants as possible and to avoid the bias introduced by having access to the internet, the questionnaire was administered online and on paper. Although all questionnaires were filled out by the participants themselves, the setting where the participant completed it (e.g., at home vs. at the hospital) could have influenced the results. Finally, a health education session was not provided after participation, which could be considered in future studies.

## Conclusion

In conclusion, patients with GC in Wuxi demonstrated poor KAP regarding nutritional support. Socioeconomic status was a significant factor affecting KAP, with lower KAP observed in patients with a lower socioeconomic status. Knowledge was found to influence attitudes and practices, while attitudes influenced practices. These findings suggest that improving patient education could enhance the KAP toward nutritional support in the population of patients with GC.

## Data Availability

The original contributions presented in the study are included in the article/Supplementary material, further inquiries can be directed to the corresponding author.

## Footnotes

^1^ https://www.wjx.cn
